# The Microbiome in Cystic Fibrosis Pulmonary Disease

**DOI:** 10.3390/genes11050536

**Published:** 2020-05-11

**Authors:** Alice Françoise, Geneviève Héry-Arnaud

**Affiliations:** 1 UMR 1078 GGB, University of Brest, Inserm, EFS, F-29200 Brest, France; alice1francoise.af@gmail.com; 2 Unité de Bactériologie, Pôle de Biologie-Pathologie, Centre Hospitalier Régional et Universitaire de Brest, Hôpital de la Cavale Blanche, Boulevard Tanguy Prigent, 29200 Brest, France

**Keywords:** cystic fibrosis, lung microbiome, metagenomics, gut–lung axis

## Abstract

Cystic fibrosis (CF) is a genetic disease with mutational changes leading to profound dysbiosis, both pulmonary and intestinal, from a very young age. This dysbiosis plays an important role in clinical manifestations, particularly in the lungs, affected by chronic infection. The range of microbiological tools has recently been enriched by metagenomics based on next-generation sequencing (NGS). Currently applied essentially in a gene-targeted manner, metagenomics has enabled very exhaustive description of bacterial communities in the CF lung niche and, to a lesser extent, the fungi. Aided by progress in bioinformatics, this now makes it possible to envisage shotgun sequencing and opens the door to other areas of the microbial world, the virome, and the archaeome, for which almost everything remains to be described in cystic fibrosis. Paradoxically, applying NGS in microbiology has seen a rebirth of bacterial culture, but in an extended manner (culturomics), which has proved to be a perfectly complementary approach to NGS. Animal models have also proved indispensable for validating microbiome pathophysiological hypotheses. Description of pathological microbiomes and correlation with clinical status and therapeutics (antibiotic therapy, cystic fibrosis transmembrane conductance regulator (CFTR) modulators) revealed the richness of microbiome data, enabling description of predictive and follow-up biomarkers. Although monogenic, CF is a multifactorial disease, and both genotype and microbiome profiles are crucial interconnected factors in disease progression. Microbiome-genome interactions are thus important to decipher.

## 1. Introduction

Gene discovery and progress in genetics and genomics have dramatically modified our view of precision medicine [1,2]. Cystic fibrosis (CF) is a monogenic disease implicating mutations of both copies of the gene coding the cystic fibrosis transmembrane conductance regulator (CFTR) protein, thus inherited in an autosomal recessive manner. The *cftr* gene has been known for more than 30 years and mutation screening for CF is now routine [3]. However, CF shows great and incompletely understood clinical heterogeneity, which wide allelic heterogeneity and functional classification of clinical mutations fail to explain. Several studies over the last five years explored *cftr* genotype–phenotype relationships [4,5], establishing that the disease depends on a balance between *cftr* mutations and the combined influence of modifier genes and other poorly characterized factors [6,7]. 

CF is thus a multifactorial monogenic disease, whose pathophysiology remains to be explained, particularly concerning infectious pulmonary disease. Chronic lung infections are the primary cause of morbidity-mortality in CF. The CF respiratory tract is colonized by numerous bacteria from an early age [8]. Despite tremendous progress, CF patients still die from lung infection. Discovering factors for airway infection could help identify mechanisms for increased susceptibility to infection, with subpopulations for aggressive screening and therapy. Many studies explored the link between cftr genotype and respiratory phenotype [9,10,11,12]. While p.F508del mutation was associated with *Pseudomonas aeruginosa* colonization [12], the most threatening microbial pathogen in CF [13], the correlations that can be established between *cftr* mutations and the progression of lung disease do not fully explain the lung phenotypes of CF patients. For example, patients with the same *cftr* genotype may have a clinical discordance, including siblings with CF [14].

Until recently, CF-related lung disease research focused on major pathogens such as *P. aeruginosa*. However, just as genetics has been interested in genes other than *cftr* [4,15], microbiology is also undergoing a paradigm shift, considering the whole microbial environment and not just one pathogen. In both fields, this shift was enabled by new technology: next-generation sequencing (NGS).

This review aims to describe the modalities and value of microbiome exploration in CF pulmonary disease, complementing genetic data. The development of metagenomics tools and of “-omics” in general provides decisive new knowledge about microbial communities associated with humans and their interactions with host and environmental factors. This review will focus mainly on describing the airways microbiome, but it will also address the gut microbiome through the gut–lung axis, which is very important to decipher the respiratory disease.

## 2. Deciphering the Microbiome

### 2.1. New Technology, New Vocabulary 

The term “microbiota” refers to all the microorganisms (bacteria, viruses, fungi, archaea, protists) present in an ecosystem [16]. It can be explored by genomic mapping of all microorganisms in the studied environment, leading to the description of the microbiome (microbi*-ome*, i.e., “*-ome*” part of the microbes) [16,17]. In microbial ecology, the term “microbiome” also refers to the entire habitat: microorganisms, their genomes, and microscopic environmental conditions (micro*-biome*) [16,17]. Complete microbiome study further includes intracellular mechanisms and interactions between microorganisms or between microorganisms and their host and environment; this is the aim of complementary approaches such as transcriptomics or metabolomics [18,19]. Disease-associated microbiome alterations are often referred to as a “dysbiosis”, a term that is widely used in the microbiome field but remains vaguely defined and is often misused. However, in chronic conditions such as CF, the term is relevant. Dysbiosis can be analyzed at different levels (taxonomic, functional), but most often it is assessed at the taxonomic level; dysbiosis is defined as the loss or gain of bacteria that promotes health or disease, respectively [18,19].

Most microbiome studies actually concern only a fraction of it: bacterial communities, but the microbiome also comprises all the genetic material provided by viruses, fungi and archaea: virome, mycobiome, and archaeome; however, data remain scant, and “microbiome” implicitly still refers to bacteria. All microbiome data are based on taxonomy enabling predictions and hypotheses based on knowledge of identical microorganisms. The most commonly used ranks, in ascending order, are species, genera, families, orders, classes, phyla, and domains (Table 1) [20,21,22,23]. With the emergence of genomics, other dimensions have been added. Operational taxonomic units (OTUs) are clusters of similar sequence variants recovered from high-throughput marker gene analysis (usually *rrs* gene that encodes bacterial 16S rRNA). Each cluster represents a taxonomic unit (species or genus depending on sequence similarity threshold and type of bacterium). Typically, a 97% 16S gene sequence identity threshold defines OTUs. Amplicon sequence variant (ASV) is a new term referring to individual DNA sequences recovered after removing spurious sequences generated during amplification and sequencing [24]. ASVs use a method resolving individual sequences without clustering. ASVs are thus inferred sequences of true biological origin. Given the high diversity of human microbiomes, simplifying methods are proposed, classifying the microbiome into clusters based on OTU abundance, first applied to the gut microbiome: three human enterotypes were described worldwide, independent of age, gender, body weight, or ethnic group, but diet-dependent in the long-term [25]. This method was then applied to other niches (pulmotypes, vaginotypes, etc.).

NGS boosted analysis of human microbial communities, but without making traditional bacterial culture redundant if throughput is high. The era of metagenomics is also the era of high-throughput culture-based approach. We will see how these two complementary approaches are practiced.

### 2.2. Molecular-Based Strategies 

#### 2.2.1. Sampling and Pre-Analytical Consideration

In CF, the two main microbiomes are gut and lung, being the most affected [26]. For the gut microbiome, most studies use stool samples, easy to collect non-invasively. In addition, feces show less eukaryotic contamination, facilitating pre-analytical processing, especially since bacterial load is high (10^11^ colony forming unit (CFU)/gram feces). Conversely, lung microbiome samples must be retrieved from the lower respiratory tract and bacterial load is lower [17]. However, pulmonary colonization density is much higher. This allows pulmonary microbiome study in sputum, where contamination is minimal in CF patients expectorating spontaneously [27,28,29]. Bronchoalveolar lavage (BAL) used to be the only method for non-expectorating patients, but induced sputum has been validated as reflecting CF bronchopulmonary bacterial communities, and is far less invasive, allowing iterative sampling for close monitoring [30,31]. 

For molecular methods, there are many points of vigilance; two must be monitored as they greatly influence outcome [26]: nucleic acid extraction, because many species are difficult to lyse, and contamination risk, as bacteria are ubiquitous, including in the DNA extraction or amplification kits (“contaminome” or “kitome”) [32].

#### 2.2.2. Targeted or Shotgun Metagenomics

The study of microbial communities in clinical niches focuses on two key questions: 

Who is there? This is addressed by ribosomal RNA gene profiling (targeted metagenomics or metagenetics) [2], resolving the richness (number of OTUs per sample), evenness (similarity of proportions of the different OTUs in a sample) and diversity (number of OTUs per sample and their abundance) of the community (bacteria, fungi) up to OTU or ASV level. For bacteria, the target is the 16S rRNA gene, common to all bacteria, with nine variable regions (V1–9) enabling taxonomic affiliation interspaced by constant regions, allowing primer hybridization. For fungi, the target is ITS1, ITS2, or 18S rRNA genes [33], and for archaea, selected 16S rRNA gene domains; however, this last domain has not been extensively studied yet [34]. Viruses lack any universal gene, precluding a targeted-metagenomic approach [35]. Choice of library preparation and sequencing method largely depends on local facilities. Illumina technology is the most widely applied worldwide in metagenomics. The MiSeq Illumina platform has short reading lengths (50–300 nt), that can be extended to 2 × 300 nt by reading amplified DNA in two directions. This technology provides only a partial view of genes, preventing taxonomic affiliation down to species level for all reads, and describing ecosystems at best at genus level. Conversely, long-read sequencing (e.g., real-time sequencing, Pacific Biosciences; nanopore sequencing, Oxford Nanopore Technologies) can determine genes’ full-length, allowing fine microbiome resolution and use of bioinformatic tools such as Picrust software, designed to predict metagenome functional content from marker genes [36]. 

What are they doing? This is addressed by whole metagenome shotgun sequencing, facilitated as high-throughput technologies become more affordable, and consisting of untargeted sequencing of all microbial genomes directly after extraction, without amplification, limiting bias induced by primers. It provides complete information whatever the microorganism (bacteria, phages, archaea, eukaryotic parasites): taxonomic composition, microbial community functional potential, and epidemiology [37]. As whole genome sequences may be reconstructed, metagenomics may elucidate community composition up to clonal complex level, reconstructing metabolic pathways [38,39]. In CF, shotgun metagenomics generated unbiased quantitative diversity data in lung, discerning more species than targeted metagenomics [35,37]. It is essential for virome study. Multiplex PCR kits detect most airway-invading viruses but do not provide quantification data or detect the entire virus population. Metagenomics offers a precious alternative for exploring the lung virome, and also the CF archaeome in years to come [35,37].

Other “-omics” approaches complete community analysis. Transcriptomics and proteomics estimate the degree of expression of previously identified genomes [26,40]. As several bacterial metabolic pathways influence many ecosystem parameters, metabolomics may extend our understanding of microbial functions in CF lung [19,41,42].

### 2.3. Culture-Based Strategy 

Studies have shown the quantitative and qualitative importance of non-cultivable or hard-to-cultivate bacteria such as anaerobes, unable to grow or even killed by oxygen. Thus, species important in the pathophysiology of bowel disease, like *Faecalibacterium prausnitzii,* were revealed by NGS [43]. Anaerobes were expected in the gut microbiome, but their level in the lungs was surprising [44]. These NGS data encouraged a return to culture, but with high throughput by multiplying culture conditions (enriched media, strict anaerobic atmosphere, extended incubation time, etc.) and systematic identification of each colony morphotype on MALDI-TOF mass spectrometry. Many improvements in culture media broaden the spectrum of cultivable bacteria. Artificial media mimic natural conditions, recreating macromolecular composition and abiotic conditions (pH, electrolytes concentration, etc.): artificial sputum mimicking bronchopulmonary mucus [45]; or creating new culture facilities: fermenters mimicking the gastrointestinal tract [46] or artificial mucus-clogged bronchiole [47]. The “culturomics” extended-culture approach can culture bacteria previously considered “uncultivable” [48]. It also explores potential microbial interactions identified in meta-genetic studies and characterizes bacterial metabolites of interest [49,50,51]. In CF, extensive culture-enriched airway microbiome profiling identified bacterial families, such as *Ruminococcaceae* or *Bacteriovoracaceae,* in CF sputa not detected by 16S rDNA sequencing alone [52].

### 2.4. Animal Models

The microbiome is very sensitive to environmental factors such as diet, antibiotics, age, sex, etc. In animal models, these confounding factors can be better controlled (although cage effects were reported) [53,54]. In CF, there are several models, with CF mouse models being the most common, although not optimal for studying pulmonary disease [55]. As previously reviewed [55,56], CF ferret [57], rabbit [58], pig [59], sheep [60], or rat [61] models could be future alternatives for the study of microbiome as they show closer anatomy or pulmonary phenotype with humans than mice. Metagenomic studies have yet to be done. Analysis of CF mouse intestinal microbiota highlighted bacterial overgrowth as well as a decrease in microbiome richness and diversity [62,63,64]. This was replicated, but dysbiosis intensity seems model-dependent [64]. 

Different conditions can be chosen for animal microbiome experiments. Antibiotics can be used to study the effect of microbiome disruption on a function of interest, for example, to test how CF patients may react to the cocktails they receive. Lynch et al. demonstrated that changes in CF and non-CF mouse microbiome under antibiotics were greater than the pre-treatment difference between the two types of mice [65]. Germ-free animals [66] or animals under different diets [67] are other ways to explore microbiome function. Finally, animal models can explore the gut–lung axis or specific microbial interactions identified as pathophysiologically critical by -omics studies [68]. A major issue is that animal and human microbiomes are of different composition; indeed, results in mice are often not seen in humans. Humanized microbiome mouse models might overcome this [56], but have not yet been applied in CF.

## 3. CF Microbiome Landscape

### 3.1. Airway Microbiome

The respiratory tract measures approximately 50–75 m^2^ and is an open door to our environment. Its anatomical diversity (trachea, bronchi, bronchioles, alveolar sacs) corresponds to pulmonary geography (biogeography) [17,19]. The main pulmonary bacterial phyla are Bacteroidetes and Firmicutes and to a lesser extent Proteobacteria and Actinobacteria [17,69]. High-throughput 16S rRNA gene sequencing highlighted a "core microbiome" of taxa present in most individuals [17,69]. In healthy subjects, it mainly comprises *Streptococcus*, *Prevotella*, *Fusobacterium*, *Veillonella*, *Porphyromonas*, *Haemophilus* and *Neisseria* [69]. Interestingly, this organ dedicated to oxygenation hosts abundant strictly anaerobic bacteria, such as *Prevotella, Fusobacterium, Veillonella,* and *Porphyromonas*. 

#### 3.1.1. CF Airways Microbiome Ecology

In CF, the absence or dysfunction of CFTR protein significantly impacts mucus rheology [70], particularly at the respiratory level, conferring hyperviscosity and promoting polymicrobial proliferation and microbial imbalance (dysbiosis) along the respiratory tract. More than 1000 species were identified in CF airways by shotgun metagenome sequencing of induced sputum [71,72]. On nasal microbiota analysis [71], while healthy subjects displayed a continuum in upper and lower airway microbiomes [73], graduated sample analysis of the CF respiratory tract (nasal, nasopharyngeal, oral, and lung samples) demonstrated dissimilarities between the two [71,74,75]. The more advanced the disease, the more pronounced the difference [74]. Analysis of CF sputum and BAL samples revealed complex microbial communities where all parts of the living microscopic domains could be described: bacterial microbiome, virome, and archaeome. 

Bacterial microbiome: The complexity of the CF pulmonary microbiome is such that classical culture cannot provide an exhaustive bacterial inventory. NGS has greatly advanced understanding of CF pathophysiology. Actinobacteria, Bacteroidetes, Firmicutes, Fusobacteria, and Proteobacteria constitute >99% of the CF airway community. The CF pulmonary microbiome shows overrepresentation of Proteobacteria and Actinobacteria [72]. The core microbiome comprises *Streptococcus, Prevotella, Veillonella, Rothia, Actinomyces, Gemella, Granulicatella, Fusobacterium, Neisseria, Atopobium*, and *Porphyromonas* [23,76,77,78], with variations in other taxa [22]. Notably, anaerobics are fewer than in non-CF lung microbiomes, which may be significant for CF pulmonary physiopathology [44]. The microbiome perspective also better deciphers the multidrug-resistance gene determinants by predicting the ecosystem “resistome” (i.e., all antibiotic-resistance genes in both pathogenic and non-pathogenic bacteria) [79].

Virome: The CF lung virome is strongly affected by the mucosal environment and impaired immunity [35]. Common respiratory viruses are found in 60% of CF patients (more than in the general population) and cause greater morbidity than in non-CF subjects [80]. Presence correlates with inflammation, as they interfere with IFN and NF-kappaB pathways, and with bacterial co-infection (including *P. aeruginosa*), inducing exacerbation and impaired lung function [35,80]. In addition to eukaryotic viruses, the CF lung microbiome contains phages adapted to this particular environment [35,72,81], and known to impact the microbiome, driving pathogen adaptation and antibiotic resistance [81].

Mycobiome: Fungi such as *Aspergillus fumigatus* are long-known opportunistic pathogens for CF patients, detected in sputum [82]. However, most CF airway fungi belong to the *Candida* or *Malassezia* families and are mostly transient [33]. Fungal species may interact with the bacteriome and/or virome and be a cofactor in inflammation and immune response [83]. Deciphering the inter-kingdom network may elucidate CF pulmonary disease [84].

Archaeome: Archaea are a group of single-cell prokaryotic organisms, previously classified as bacteria but now distinguished in a specific domain, beside bacteria and eukaryotes. They are found in anaerobic environments, including human. Exploration of human-associated archaea is still very new but has demonstrated diversity between anatomical niches [85]. In the CF lung, all archaeal phyla show <0.1% abundance [72,85]. 

#### 3.1.2. CF Airway Microbiome Dynamics Throughout Disease Course

Disease course can be seen through the prism of the lung microbiome. Microbiome progression is individual-specific, requiring personalized medical follow-up [20]; nevertheless, trends emerge. The intestinal microenvironment predisposes young CF children to intestinal and respiratory dysbiosis, possibly from birth [8]. Up to 11 years of age, microbial diversity is high [86], then, as *P. aeruginosa* colonization becomes chronic, richness and diversity is lost with age, disease progression, and dominance of pathogens [29,87]. Diversity is a marker of lung function. In long-term follow-up (10 years), diversity was maintained in patients with stable respiratory function, and decreased in patients with impaired function on FEV1 [87]. This decrease correlates with the establishment of a dominant pathogen [88], usually *P. aeruginosa,* whose prevalence increases with age [20,22]. Other taxa associated with CF pathogenicity (*Staphylococcus*, *Haemophilus*, *Burkholderia*) are also more prevalent in older patients [20,74]. Other pathogens of increasing concern (non-fermentative Gram-negative bacilli: *Achromobacter, Stenotrophomonas*) show similar colonization patterns, leading to persistent infection [89].

Variations in microbiome profile were also described in patients with pulmonary exacerbations (PEx). *P. aeruginosa* or other pathogens are systematic in sputum of CF PEx patients [90], but anaerobes are key components in PEx [91]. Variations in several anaerobic genera (e.g., *Prevotella*) account more for variability in respiratory function after treatment and in the metabolic environmental shift during PEx than the dominant opportunistic genera *Pseudomonas* [40,90]; thus, anaerobes may be better PEx biomarkers than the commonly used diversity, which shows no difference or diminution [20,92]. Long-course antibiotics also impact microbiome maturation and evolution. During exacerbations, antibiotherapy modulates the microbiome, decreasing diversity and richness; long-term effects include reduced commensal bacterial population not corrected after wash-out [92]. 

### 3.2. Gut–Lung Connection

CF gut–lung dialogue is interesting, as gut and lung microbiomes are disrupted by the same etiology (loss of CFTR function), making their interactions more complex. Dysbiosis of the two sites is partially independent. Altered microbial communities in gut and lung is governed by organ-specific micro-environmental conditions (viscous mucus, hyperinflammation, etc.). However, the two microbiomes also interact. The intestinal microbiome especially impacts pulmonary microbiome constitution via microbial metabolite exchange [69]. In CF, the gut–lung axis is disrupted by decreased abundance of bacteria producing short-chain fatty acids (SCFAs) [93,94], which have immunomodulatory properties, so that the gut dysbiosis correlates with pulmonary immune homeostasis defects [93,94]. Close interaction between intestinal and pulmonary microbiotes was shown in a murine CF model; Bazett et al. [63] revealed pulmonary hyper-reactivity in response to antibiotic-induced intestinal dysbiosis. Therefore, loss of gut microbiome diversity and functional potential under repeated antibiotic treatment (often started at an early age) may exacerbate pulmonary disease in CF patients [95].

## 4. Deciphering Genome–Microbiome Interactions

### 4.1. Influence of Cftr Mutation on Pulmotypes and Enterotypes

Human genetic variation is a factor in interpersonal differences in microbiomes. Genes directly influence health by promoting a beneficial microbiome [96]. Studies of intestinal microbiome heritability revealed a subset of microbes whose abundance is partly genetically determined by the host [97]. Microbiomes are more similar for monozygotic twins than for dizygotic twins [97]. One of the most hereditary taxa is the *Christensenellaceae*, a family of bacteria that has been shown to promote a lean host phenotype. It is estimated that the host genotype influences 30–60% of the variation in the relative abundance of *Christensenellaceae* [97]. However, genome-wide association studies to identify human genetic variants associated with microbiome phenotypes is proving difficult. What about cystic fibrosis?

In mice, loss of *cftr* gene function causes intestinal dysbiosis. A close relationship was demonstrated between *cftr* genotype and microbiome constitution [66]. CF mice initially germ-free and transplanted with fecal microbiota from non-CF mice had a different microbiological profile than non-CF controls [66]. However, the exact mechanisms of microorganism selection by genotype are unknown. In humans too, CFTR protein functional impairment alters the gut microbiome [98]. Studies of the link between the type of *cftr* mutation and effect on the microbiome showed conflicting results. Microbiomes differed depending on whether the patient had one or two alleles with p.F508del mutation or else two alleles with other mutations [11,95], but further analyses found no such significant differences [99,100]. This may be explained by two factors. The first is the possible involvement of many modulator genes, in addition to the *cftr* gene, in microbial community selection [9,101]. In CF gut samples, abundance of Actinobacteria depends on the number of p.F508del alleles, but the *cftr* mutation profile does not explain the modulated bacterial metabolic pathways whereas more than 1000 genes can be otherwise over- or under-expressed [9]. The second factor is the mutual influence of genotypes and microbiome patterns (e.g., enterotypes for the gut microbiome and pulmotypes for the lung microbiome). Microbiome disruption, by antibiotics [101] or diet [67], also affects the level of expression of essential intestinal genes and even CF modifier genes such as *Slc6a14* [101]. 

### 4.2. Effects of CFTR-Modulating Therapies on the Microbiome

CFTR modulators, including ivacaftor, have CFTR-dependent and CFTR-independent effects on the microbiome [102]. In the intestinal microbiome [103], ivacaftor increases *Akkermansia*, a beneficial bacterium involved in mucosal protection, and decreases *Enterobacteriaceae,* which correlates with decreased fecal calprotectin, an inflammation marker. In the lung microbiome, significant positive changes occurred within 48 h of initiation of ivacaftor and lasted for the first year; it reduced relative abundance of *Pseudomonas* [104,105], and increased relative abundance of endogenous species (*Streptococcus*, anaerobes) [76]. This shift to a more diverse microbiome is the hallmark of a “healthier” CF microbiome. Studies showed a negative association between microbial diversity and respiratory tract inflammation [20], and positive correlation between increased taxa count and FEV1 [64]. However, neither gut nor lung microbiome changes were sustained in the second year [76,105,106,107]. 

## 5. Toward A New Microbiome-Based Medicine

### 5.1. A Source of New Prognosis and Diagnosis Biomarkers 

Global microbiome parameters such as diversity, richness, or dominant populations are potential prognostic factors to be monitored [108,109,110]. Microbiome diversity in particular is a major predictive marker of disease progression in young adults, correlating with risk of subsequent lung transplantation and death [109]. In a decade-long study of the CF lung microbiome, community diversity decreased significantly over time in patients with typically progressive lung disease but remained relatively stable in mild lung disease phenotypes [87]. This rethinking of CF-associated airway infection in the light of microbiome analysis may be useful for clinicians making the often complicated decision about what antibiotic(s) to use in these complex infections [111]. That is the goal of the CF-MATTERS study, the first randomized controlled trial to compare microbiome-directed versus standard antibiotic therapy for CF patients with respiratory infections (https://www.cfmatters.eu/).

Similarly, clinical trial designs may need a baseline microbiome study to stratify patients according to dominant microbe. The efficacy of inhaled aztreonam, an antibiotic targeting *P. aeruginosa* in the CF airway, was evaluated using alternative outcomes according to microbiome effect [112]; benefit depended essentially on impact on species other than *P. aeruginosa*.

In the era of predictive medicine, the microbiome may be a source of new biomarkers for follow-up and early intervention. Risk of *P. aeruginosa* early colonization may be assessed on predictive biomarkers within the microbiome. *Porphyromonas* is a candidate biomarker in the lungs (BEACH study; ClinicalTrials.gov Identifier: NCT03947957) [78], and *Parabacteroides* in the gut [8]. The predictive potential of the microbiome for exacerbation was studied to adapt antibiotic therapeutic strategies. Three genera (*Streptococcus, Haemophilus, Staphylococcus*) emerged as predictive markers of antibiotic response [112].

### 5.2. A Source of Innovative Therapies

#### 5.2.1. Identification of Beneficial Microbes

Identifying potentially beneficial bacteria in CF consists first in comparing patients’ microbiomes versus healthy subjects to detect significant differences in abundance of well-known beneficial microbes such as *Bifidobacterium* or *Lactobacillus*, or new-generation probiotics such as *F. prausnitzii* [98]. For the lung microbiome, larger genetic screening is needed, as the concept of lung probiotics is new, and beneficial microbes maybe different from those in the gut [78,113,114]. Candidate probiotic properties must then be confirmed in vitro and in vivo. The immuno-modulatory potential of *Bacteroides* from CF patients has been assessed in vitro [115], which should be followed by in vivo safety and efficacy experiments [116,117,118]. 

#### 5.2.2. Other Innovative Therapies for the Gut Microbiota 

In the gut–lung axis, dietary involvement offers a microbiome-based therapeutic perspective for preventing lung disease by manipulating the gut microbiome. Diet fortification with certain fatty acids [119] or carbohydrates is of interest, as these regulate production of SCFAs, which have a positive impact on lung function in CF patients [93,120]. Vitamin D supplementation, essential for the development of a healthy intestinal microbiota, could also be beneficial for patients who are generally deficient due to malabsorption and dysbiosis [121]. Ultimately, knowledge acquired on the “gut–lung” axis may guide fecal microbiota transplantation in respiratory pathologies; only randomized controlled trials can enable progress on this therapeutic track.

## 6. Conclusions

**Figure 1:**
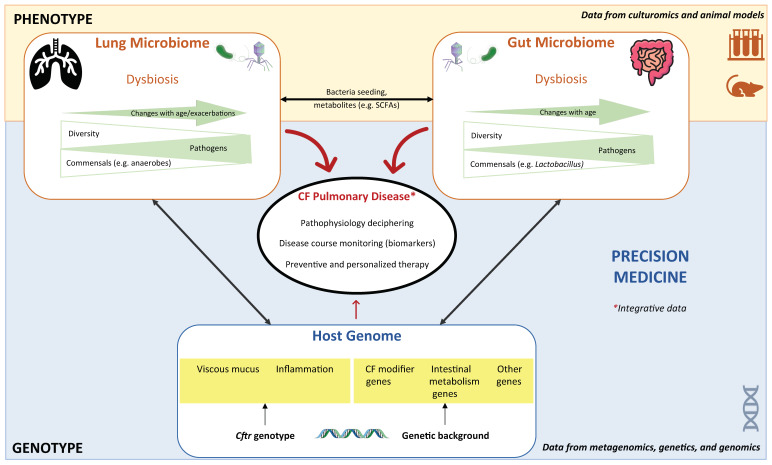
Contribution of microbiome science to cystic fibrosis (CF) research. This figure summarizes the interplay/complementarity between metagenomics and genetics in deciphering CF lung disease, and the combined tools in microbiome research. The genotype profile is stable and fixed since birth, whereas the phenotype provided by the microbiome profiles from both niches, lungs and gut changes with age. Both sets of data are necessary for precision medicine in CF.

In conclusion, in the era of NGS, it seems just as fundamental to establish the microbial profile of a CF patient as to establish his/her genotype in order to understand the unique disease progression of each patient, particularly in respiratory sites. Although essentially based on DNA analysis, the microbiome provides the indispensable complement to interpret genotype: the phenotype. The microbiome comprises an extremely rich sum of data, enabling precise individual assessment, and is now an essential key to improving precision in CF management by providing prognostic and monitoring biomarkers, and possibly innovative therapeutic solutions. In the future, machine learning integrating data from the joint efforts of geneticists and microbiologists will be crucial for better understanding of this infectious genetic disease (Figure 1).

## Figures and Tables

**Table 1:** Principal taxa in gut and lung cystic fibrosis (CF) microbiomes, presented according to bacterial taxonomy [20,21,22,23].

	Domain	Phylum	Class	Order	Family	Genus	Species
**CF lung** **microbiome**	Bacteria	Bacteroidetes	Bacteroidia	Bacteroidales	*Prevotellaceae*	*Prevotella*	*P. denticola*
						*Porphyromonas*	*P. catoniae*
		Firmicutes	Bacilli	Lactobacillales	*Streptococcaceae*	*Streptococcus*	*S. oralis*
						*Granulicatella*	*G.adiacens*
						*Gemella*	*G. haemolysans*
						*Staphylococcus*	*S. aureus*
						*Veillonella*	*V. parvula*
		Proteobacteria	Gammaproteobacteria	Pseudomonales	*Pseudomonadaceae*	*Pseudomonas*	*P. aeruginosa*
						*Burkholderia*	*S. maltophilia*
						*Achromobacter*	*B. cenocepacia*
						*Stenotrophomonas*	*A. xylosoxidans*
						*Neisseria*	*N. mucosa*
						*Haemophilus*	*H. influenzae*
		Actinobacteria	Actinobacteria	Actinomycetales	*Actinomycetaceae*	*Actinomyces*	*A. odontolyticus*
						*Rothia*	*R. mucilaginosa*
						*Atopobium*	*A. parvulum*
		Fusobacteria	Fusobacteriia	Fusobacteriales	*Fusobacteriaceae*	*Fusobacterium*	*F. nucleatum*
**CF gut** **microbiome**	Bacteria	Bacteroidetes	Bacteroidia	Bacteroidales	*Tannerellaceae*	*Parabacteroides*	*P. distasonis*
						*Prevotella*	*P. coprii*
						*Veillonella*	*V. parvula*
						*Bacteroides*	*B. fragilis*
		Firmicutes	Clostridia	Clostridiales	*Ruminococcaceae*	*Faecalibacterium*	*F. prausnitzii*
						*Blautia*	*B. faecis*
		Actinobacteria	Actinobacteria	Bifidobacteriales	*Bifidobacteriaceae*	*Bifidobacterium*	*B. longum*

Class, order, and family are mentioned only for the first species listed for each phylum.
